# Associations between circulating amino acids and metabolic dysfunction‐associated steatotic liver disease in individuals living with severe obesity

**DOI:** 10.14814/phy2.70171

**Published:** 2025-01-27

**Authors:** Ina Maltais‐Payette, Jérôme Bourgault, Marie‐Frédérique Gauthier, Laurent Biertho, Simon Marceau, François Julien, Patricia L. Mitchell, Christian Couture, Francis Brière, Jacques Corbeil, Benoit J. Arsenault, André Tchernof

**Affiliations:** ^1^ Quebec Heart and Lung Institute – Laval University Quebec Quebec Canada; ^2^ School of Nutrition, Faculty of Agriculture and Food Science Laval University Quebec Quebec Canada; ^3^ Department of Molecular Medicine, Faculty of Medicine Laval University Quebec Quebec Canada; ^4^ Department of Surgery, Faculty of Medicine Laval University Quebec Quebec Canada; ^5^ Laval University Medical Center – Laval University Quebec Quebec Canada

**Keywords:** amino acids, metabolic‐dysfunction associated steatotic liver disease, obesity

## Abstract

Metabolic dysfunction‐associated steatotic liver disease (MASLD) describes liver diseases caused by the accumulation of triglycerides in hepatocytes (steatosis) as well as the resulting inflammation and fibrosis. Previous studies have demonstrated that accumulation of fat in visceral adipose tissue compartments and the liver is associated with alterations in the circulating levels of some amino acids, notably glutamate. This study aimed to investigate the associations between circulating amino acids, particularly glutamate, and MASLD. In addition, we hypothesized that liver steatosis, concomitant with visceral adiposity, could contribute to the association between circulating glutamate and visceral obesity. We studied a sample of 150 patients living with severe obesity who were non‐diabetic and selected to represent a wide range of MASLD severity. Liver histological features were determined by a pathologist from a biopsy sample obtained at the time of bariatric surgery. Bulk RNA sequencing measured the hepatic mRNA expression level of selected genes related to the urea cycle and glutamate metabolism. Fasting plasma amino acid levels were measured by liquid chromatography coupled with tandem mass spectrometry. Patients with more advanced steatosis had larger visceral adipocytes, higher levels of circulating tyrosine, glutamate, and alanine as well as lower levels of serine. MASLD severity was significantly associated with the hepatic mRNA expression of glutamate metabolism genes such as GLS1, GLUL (positively), and NAGS (inversely). In individuals living with obesity, MASLD severity is associated with visceral adipocyte hypertrophy, higher circulating glutamate as well as potential alterations of hepatic amino acid and nitrogen metabolism.

## 1 INTRODUCTION

In the context of a positive energy imbalance, the limited expansion capacity of the subcutaneous fat compartments progressively leads to increased reliance on lipid storage within anatomical structures such as the greater omentum and mesentery, termed visceral obesity, as well as ectopic sites such as the liver (Neeland et al., 2019). Liver steatosis is the accumulation of lipid droplets in at least 5% of hepatocytes (Brunt et al., 1999). Metabolic dysfunction‐associated steatotic liver disease (MASLD) is an umbrella term to describe patients who present liver steatosis and at least one cardiometabolic alteration (Rinella et al., 2023). Metabolic dysfunction‐associated steatohepatitis (MASH) is a more severe form of MASLD where hepatic fat accumulation is accompanied by inflammation, cell death, and sometimes fibrosis. These new terms have been chosen by three large pan‐national liver associations to replace non‐alcoholic fatty liver disease (NAFLD) and non‐alcoholic steatohepatitis (NASH) (Rinella et al., 2023).

Many studies have highlighted that cardiometabolic diseases are associated with an altered circulating amino acid profile (Newgard, 2017). Circulating concentrations of the branched‐chain amino acids (BCAAs, namely leucine, isoleucine, and valine) have been repeatedly shown to be elevated in individuals with obesity and to predict type 2 diabetes (T2D) risk (Newgard, 2017). Our group and others have previously demonstrated that plasma glutamate level is the amino acid most strongly associated with abdominal and visceral obesity (Kimberly et al., 2017; Maltais‐Payette et al., 2018, 2019, 2022; Yamakado et al., 2012). The pathophysiological mechanisms underlying this association have yet to be elucidated.

Among the studies reporting associations between MASLD and circulating amino acids, the most common results are a positive association with isoleucine, leucine, valine, and tyrosine and a negative association with glycine (Tricò et al., 2021). In the healthy state, circulating glucagon can stimulate hepatic amino acid catabolism by activating the urea cycle (Holst et al., 2017). In return, circulating amino acids stimulate the synthesis and secretion of glucagon by alpha cells in the pancreas, creating a feedback loop called the liver‐alpha cell axis (Holst et al., 2017). Studies have suggested that this phenomenon is altered in the context of liver steatosis (Galsgaard, 2020) and that these alterations could explain the association between circulating amino acids and MASLD. However, most of the studies on this topic did not account for adiposity and T2D status, which are associated with an altered amino acid profile and MASLD severity. Therefore, it is currently challenging to decipher which changes in circulating amino acids or liver‐alpha cell axis are specific to liver alterations. Moreover, MASLD is usually studied as a whole, without differentiating steatosis, MASH, and fibrosis. The present study aimed to investigate the association between circulating amino acids, in particular glutamate, and MASLD components. We hypothesized that impairment of the liver‐alpha cell axis in the context of MASLD contributes to the association between circulating glutamate and visceral obesity.

## 2 MATERIALS AND METHODS

### 2.1 Sample

We studied a sample of 150 bariatric surgery patients retrospectively chosen from the Institut Universitaire de Cardiologie et de Pneumologie de Québec (IUCPQ) Biobank. Patients were selected to be non‐diabetic and to present a wide range of MASLD severity, with 30 patients in each of the following study groups: (i) steatosis grade 0; (ii) steatosis grade 1 without MASH (1w/oM); (iii) steatosis grade 1 with MASH (1wM); (iv) steatosis grade 2 with or without MASH; and (v) steatosis grade 3 with or without MASH.

Groups were matched for age, sex, and preoperative body mass index (BMI). At the preoperative visit, anthropometric measurements were obtained, blood pressure was measured, and fasting blood was drawn. Hemoglobin A1c, glycemia, insulin, and lipid profile were assessed, and the homeostatic model assessment of insulin resistance (HOMA‐IR) index was calculated. Plasma glucagon was measured by sandwich enzyme‐linked immunosorbent assay (Mercodia, Uppsala, Sweden). During surgery, a liver biopsy was performed, and a trained pathologist examined part of the resulting sample to determine steatosis, inflammation, and fibrosis features. Part of the sample was flash‐frozen in the operating room at the time of sampling. Subcutaneous and visceral (greater omentum) adipose tissue samples were also obtained during surgery and immediately flash‐frozen in the operating room.

Tissue specimens and clinical data were obtained from the IUCPQ Biobank according to institutionally approved management modalities. This study was conducted in accordance with the declarations of Helsinki and Istanbul. Ethics approval was received on August 24th, 2020 (approval number 2021–3398, 21,925). All participants provided written informed consent.

### 2.2 Amino acid measurement

Circulating amino acid concentrations were measured in plasma by liquid chromatography coupled with tandem mass spectrometry using the EZ:faast kit (Phenomenex, 2003, Torrance, CA, USA) as described elsewhere (Gobeil et al., 2022). Briefly, plasma samples were extracted by solid‐phase support extraction, derivatized, and purified using a liquid–liquid extraction. Data acquisition was performed on a Waters Acquity I‐Class UPLC coupled with a Waters Synapt G2‐Si mass spectrometer. Quantification was done by comparison with an internal standard.

### 2.3 Adipose tissue characterization

Adipose tissue samples were fixed in formalin and circulated in paraffin. Microscopic slides of subcutaneous and visceral adipose tissue from each patient were prepared. For mean adipocyte size determination, slides were stained with hematoxylin and eosin, and the diameter of at least 100 fully visible adipocytes per slide was measured using ImageJ (Laforest et al., 2018).

### 2.4 Hepatic expression levels of genes involved in amino acid metabolism

Liver mRNA expression was measured by RNA sequencing in 104 participants. The detailed method can be found in (Gobeil et al., 2024). Briefly, RNA was extracted from frozen liver biopsy samples using the Qiagen RNeasy Plus kit (catalogue #74134). We used BioTech Biodrop to determine the concentration and the purity of RNA and BioAnalyser or RNA agarose gel to determine its quality. Reverse transcription was done using the NEBNext RNA First Strand Synthesis (catalogue #E7771S) and NEBNext Ultra Directional RNA Second Strand Synthesis Modules (catalogue #E7550L, New England BioLabs). cDNA was sequenced on an Illumina NovaSeq S4. The reads were mapped to the human reference genome GRCh38/hg38 with STAR v2.5.1. Relative gene expression was expressed as transcript per million, as produced by RNA‐seQC v2.4.2.

Because this project was focused on amino acids, and particularly on circulating glutamate, we followed a hypothesis‐driven approach and investigated 10 transcripts encoding proteins implicated in the urea cycle (CPS1, OTC, ASS1, ASL, and ARG1) and/or glutamate metabolism (NAGS, GLS1, GLS2, GLUL, and GLUD1).

### 2.5 Statistical analyses

Study groups were compared using the Kruskal–Wallis non‐parametric test followed by the Dunn test for multiple comparison analysis. Associations between circulating amino acids and metabolic variables were computed using Spearman rank correlations. All statistical analyses were done using R Statistical Software (v4.1.2) (R Core Team, 2021).

## 3 RESULTS

### 3.1 Sample characteristics

**FIGURE 1:**
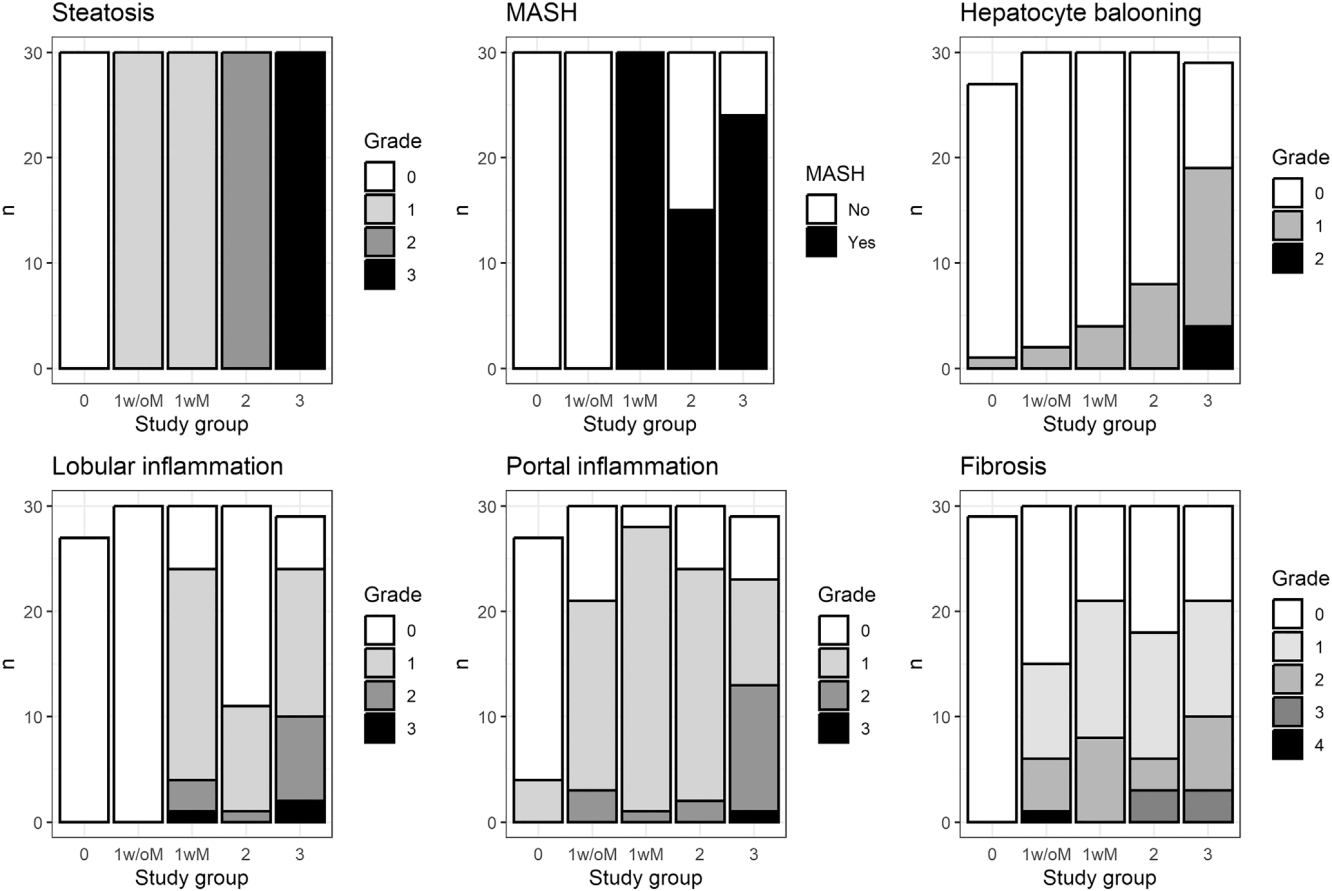
Hepatic histology by study group. Hepatic biopsy samples were obtained during bariatric surgery and examined by a trained pathologist. Grading was done according to the Brunt scoring system (Brunt et al., 1999). Study groups: 0: No steatosis, 1w/oM: Steatosis grade 1 without metabolic dysfunction‐associated steatohepatitis (MASH), 1wM: Steatosis grade 1 with MASH, 2: Steatosis grade 2 with or without MASH, 3: Steatosis grade 3 with or without MASH.

Sample characteristics are presented by study group in Table 1. As per the study design, age, sex, and BMI were not significantly different among groups. Participants with more advanced MASLD had greater neck and waist circumferences as well as higher plasma triglycerides. Although none of the patients had T2D, hemoglobin A1c and HOMA‐IR index were higher with increasing MASLD severity. The detailed liver histology features of participants are presented in Figure 1. Steatosis grade, presence of MASH, hepatocyte ballooning, lobular inflammation, portal inflammation, and fibrosis were all significantly different across groups (all *p* < 0.05).

**TABLE 1:** Sample characteristics by MASLD severity.

	*n*	Grade 0	Grade 1w/oM	Grade 1wM	Grade 2	Grade 3	*p*‐Value
Age (years)	150	37.0 (32.5–44.0)	38.0 (29.0–47.8)	37.5 (32.0–45.0)	37.5 (32.0–40.8)	36.5 (33.2–38.0)	0.9815
Sex (female)	150	25 (83%)	21 (70%)	21 (70%)	21 (70%)	25 (83%)	0.4864
BMI (kg/m^2^)	150	43.6 (41.3–50.0)	47.4 (43.7–52.5)	47.8 (44.7–51.5)	47.3 (43.8–52.7)	46.9 (44.5–57.2)	0.0727
WC (cm)	140	123 (119–139)	136 (127–152)	134 (128–143)	139 (125–149)	138 (132–147)	0.0060
NC (cm)	83	42.0 (38.5–45.0)	50.0 (47.0–52.0)	46.0 (43.0–48.0)	45.0 (43.5–47.0)	46.0 (43.5–48.0)	0.0074
HDL‐C (mmol/L)	150	1.10 (0.98–1.39)	1.21 (0.96–1.41)	1.10 (1.00–1.27)	1.10 (0.98–1.31)	1.11 (1.01–1.33)	0.9559
LDL‐C (mmol/L)	150	2.70 (2.29–3.16)	2.42 (2.04–2.85)	2.72 (2.21–3.15)	2.62 (2.25–3.18)	2.54 (2.01–3.30)	0.7795
TG (mmol/L)	150	1.21 (0.88–1.57)	1.29 (0.98–1.71)	1.60 (1.04–1.95)	1.59 (1.37–2.13)	2.06 (1.54–2.45)	3e‐04
HbA1c (%)	150	5.40 (5.23–5.50)	5.60 (5.50–5.70)	5.45 (5.30–5.58)	5.45 (5.23–5.58)	5.50 (5.40–5.68)	0.0056
HOMA‐IR index	147	4.00 (2.97–5.29)	4.62 (3.50–6.99)	5.83 (4.32–8.30)	5.98 (4.08–9.29)	6.72 (5.21–9.41)	6e‐04
Glucagon (pmol/L)	122	4.28 (3.51–5.93)	4.05 (2.04–7.33)	5.27 (3.92–8.05)	4.17 (3.70–8.53)	4.83 (2.81–7.89)	0.5828
SBP (mmHg)	150	128 (120–138)	134 (126–141)	130 (119–143)	135 (123–149)	134 (119–142)	0.4937
DBP (mmHg)	150	80.5 (75.0–84.8)	84.0 (81.0–85.8)	79.5 (75.0–86.0)	84.0 (78.5–90.0)	80.0 (76.2–84.0)	0.2576
Steatosis (%)	150	0.0 (0.0–0.0)	5.0 (2.0–18.8)	10.0 (10.0–23.8)	40.0 (40.0–50.0)	80.0 (71.2–90.0)	1e‐27

*Note*: Variables are presented as median (Q1–Q3) for continuous variables and *n* (%) for ordinal variables. Variables were compared across groups using Kruskal–Wallis test. Grade 0: no steatosis, grade 1w/oM: steatosis grade 1 without metabolic dysfunction‐associated steatohepatitis (MASH), grade 1wM: steatosis grade 1 with MASH, grade 2: steatosis grade 2 with or without MASH, grade 3: steatosis grade 3 with or without MASH.

Abbreviations: BMI, body mass index; DBP, diastolic blood pressure; HbA1c, glycated hemoglobin; HDL‐C, high‐density lipoprotein cholesterol; HOMA‐IR, homeostatic model assessment of insulin resistance; LDL‐C, low density‐lipoprotein cholesterol; NC, neck circumference; SBP, systolic blood pressure; TG, triglycerides; WC, waist circumference.

**FIGURE 2:**
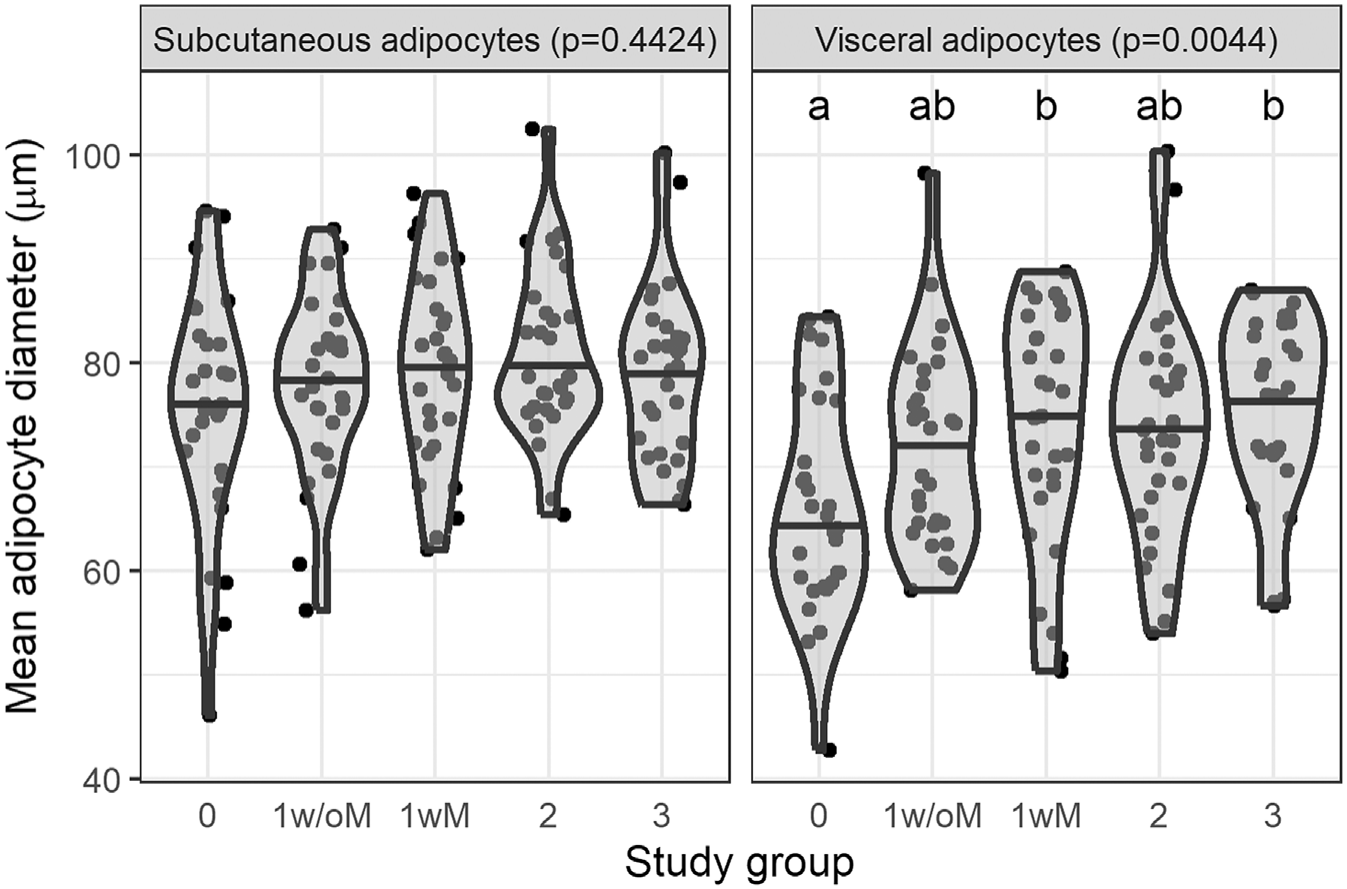
Mean adipocyte diameter in visceral and subcutaneous adipose tissue depots by study group. Variables were compared across groups using Kruskal–Wallis test, followed by Dunn's test for multiple comparison analysis. *p*‐values in the panel heading is from Kruskal–Wallis test and the letters above each group are from Dunn's test; groups that do not share a letter are significantly different. Violin plots are used to visualize the distribution of the variable and the horizontal black line represents the median by group. Adipose tissue samples were obtained during bariatric surgery and immediately flash frozen. The average size of adipocytes was measured using a histology‐based method (Laforest et al., 2018). Study groups: 0: No steatosis, 1w/oM: Steatosis grade 1 without metabolic dysfunction‐associated steatohepatitis (MASH), 1wM: Steatosis grade 1 with MASH, 2: Steatosis grade 2 with or without MASH, 3: Steatosis grade 3 with or without MASH.

As shown in Figure 2, the mean size of visceral adipocytes, but not subcutaneous adipocytes, was significantly higher with increasing MASLD severity. For example, compared to participants with healthy livers (steatosis grade 0), visceral adipocyte size was greater on average for those with steatosis grade 1 with MASH (1wM, *p* = 0.0213) and those with steatosis grade 3 (*p* = 0.0038).

### 3.2 Amino acids

**FIGURE 3:**
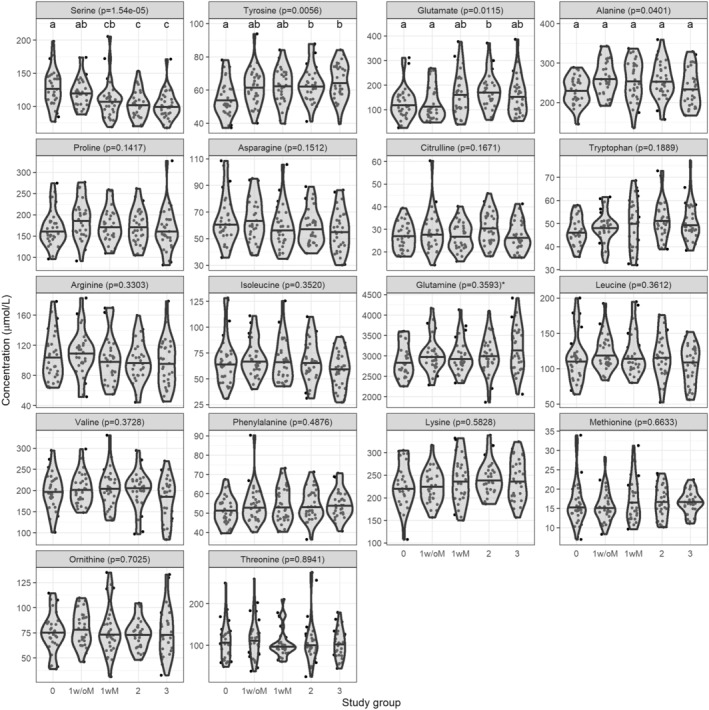
Circulating amino acids by study group. Variables were compared across groups using Kruskal‐Wallis test, followed by Dunn's test for multiple comparison analysis. *p*‐values in the panel heading is from Kruskal–Wallis test and the letters above each group are from Dunn's test; groups that do not share a letter are significantly different. Violin plots are used to visualize the distribution of the variable and the horizontal black line represents the median by group. Amino acids were measured in plasma collected while patients were in the fasted state using the EZ:Faast kit (Phenomenex, 2003, Torrance, CA, USA). Concentrations are reported as absolute, *except for glutamine which is reported as relative concentration. Study groups: 0: No steatosis, 1w/oM: Steatosis grade 1 without metabolic dysfunction‐associated steatohepatitis (MASH), 1wM: Steatosis grade 1 with MASH, 2: Steatosis grade 2 with or without MASH, 3: Steatosis grade 3 with or without MASH.

Of the 18 amino acid concentrations measured, 4 were significantly different across study groups: alanine, glutamate, and tyrosine were higher in participants with increasing MASLD severity whereas serine was lower (Figure 3). There was no significant difference in circulating amino acid levels between the patients with grade 1 steatosis with or without MASH (study groups 1w/oM vs. 1wM). The 3 BCAAs (leucine, isoleucine, and valine) were not significantly different according to MASLD severity. Although glutamine was not significantly different across groups, the glutamine‐to‐glutamate ratio was lower with increasing MASLD severity (*p* = 0.0350, results not shown).

**FIGURE 4:**
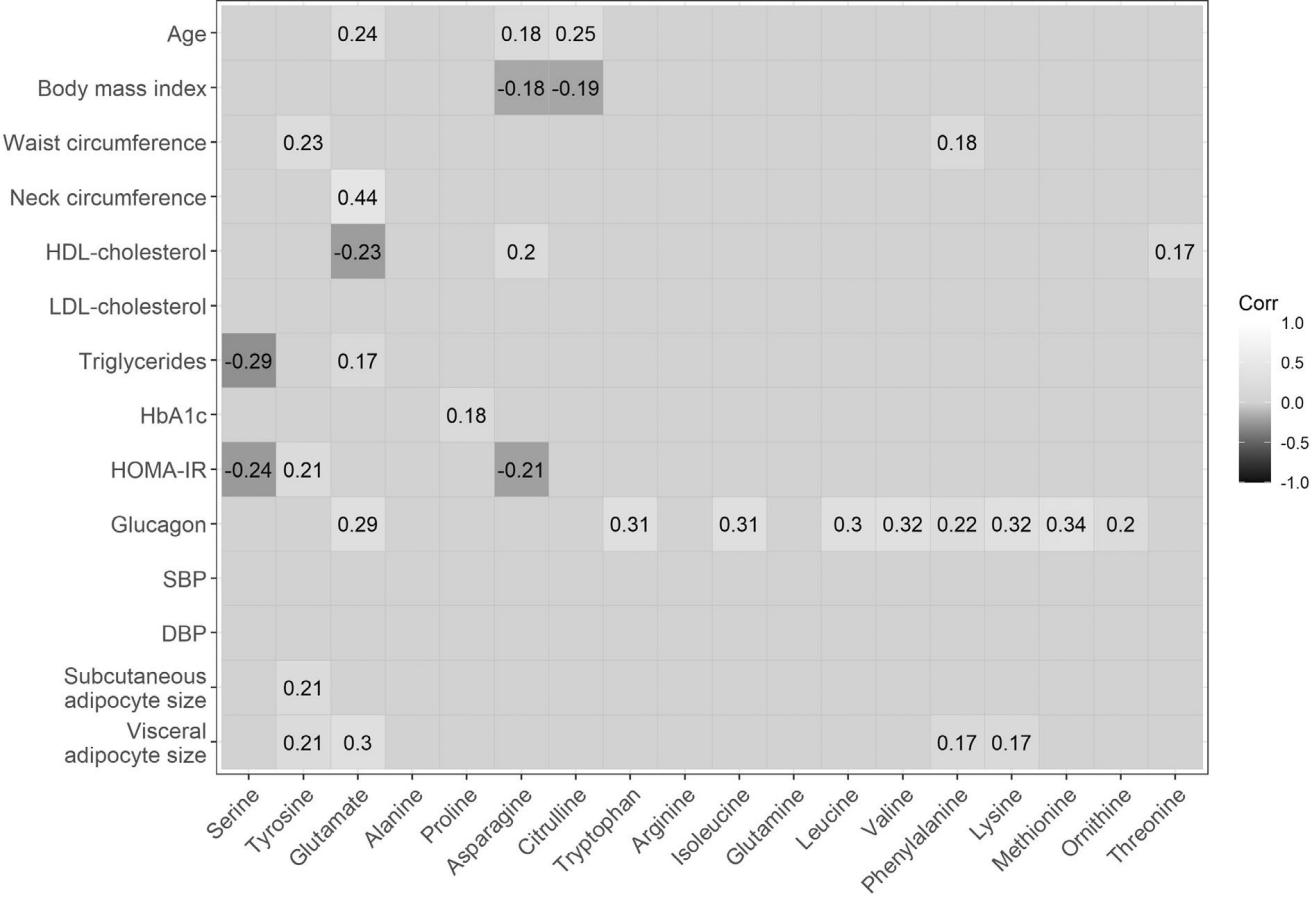
Correlations between circulating amino acid concentrations and clinical variables as well as mean subcutaneous or visceral adipocyte size. Correlations are Spearman's rho and only significant associations (*p* < 0.05) are shown. DBP, diastolic blood pressure; HbA1c, glycated hemoglobin; HDL‐cholesterol, Hhigh‐density‐lipoprotein cholesterol; HOMA‐IR, homeostatic model assessment of insulin resistance; LDL‐cholesterol, low‐density‐lipoprotein cholesterol; SBP, systolic blood pressure.

Figure 4 shows the significant correlations between circulating amino acid concentrations and clinical variables. Circulating serine was inversely associated with triglyceride concentrations and the HOMA‐IR index; circulating tyrosine was positively associated with waist circumference, the HOMA‐IR index, and the mean size of both subcutaneous and visceral adipocytes, whereas circulating glutamate was positively associated with age, neck circumference, levels of triglycerides, and glucagon as well as the mean size of visceral adipocytes, while also being inversely associated with high‐density lipoprotein‐cholesterol concentrations. In addition to glutamate, glucagon concentration was also positively associated with circulating levels of tryptophan, isoleucine, leucine, valine, phenylalanine, lysine, methionine, and ornithine.

### 3.3 Hepatic gene expression

**FIGURE 5:**
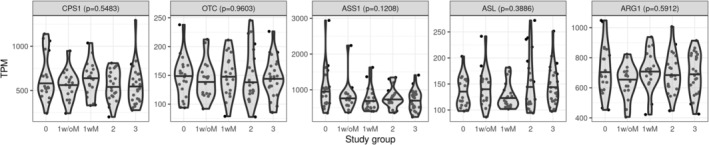
Hepatic expression of urea cycle genes by study group. Data available for 104 participants. Variables were compared across groups using Kruskal–Wallis test, followed by Dunn's test for multiple comparison analysis. *p*‐values in the panel heading are from Kruskal–Wallis test and the letters above each group are from Dunn's test; groups that do not share a letter are significantly different. Violin plots are used to visualize the distribution of the variable and the horizontal black line represents the median by group. Gene expression was measured by Illumina as part of a more extensive transcriptomics profile. Expression is reported as transcript per million (TPM). Study groups: 0: No steatosis, 1w/oM: Steatosis grade 1 without metabolic dysfunction‐associated steatohepatitis (MASH), 1wM: Steatosis grade 1 with MASH, 2: Steatosis grade 2 with or without MASH, 3: Steatosis grade 3 with or without MASH. ARG1, arginase 1; ASL, arginosuccinate lyase; ASS1, arginosuccinate synthase 1; CPS1, carbomoyl‐phosphate synthetase 1; OTC, ornithine transcarbamylase.

**FIGURE 6:**
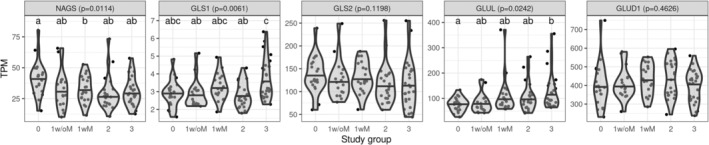
Hepatic expression of selected glutamate metabolism genes by study group. Data available for 104 participants. Variables were compared across groups using Kruskal–Wallis test, followed by Dunn's test for multiple comparison analysis. *p*‐values in the panel heading are from Kruskal–Wallis test and the letters above each group are from Dunn's test; groups that do not share a letter are significantly different. Violin plots are used to visualize the distribution of the variable and the horizontal black line represents the median by group. Gene expression was measured by Illumina as part of a more extensive transcriptomics profile. Expression is reported as transcript per million (TPM). Study groups: 0: No steatosis, 1w/oM: Steatosis grade 1 without metabolic dysfunction‐associated steatohepatitis (MASH), 1wM: Steatosis grade 1 with MASH, 2: Steatosis grade 2 with or without MASH, 3: Steatosis grade 3 with or without MASH. GLS1, glutaminase 1; GLS2, glutaminase 2; GLUD1, glutamate dehydrogenase; GLUL, glutamine synthetase; NAGS, N‐acetyl glutamate synthase.

We compared hepatic mRNA expression of genes involved in the urea cycle (Figure 5) and glutamate metabolism (Figure 6) between study groups. The hepatic expression level of N‐acetyl glutamate synthase (NAGS) was lower with increasing MASLD severity. N‐acetyl glutamate is the obligatory activator of carbamoyl‐phosphate synthetase (CPS1), the enzyme catalyzing the rate‐limiting step of the urea cycle. On the other hand, the expression of glutaminase 1 (GLS1) and glutamine synthetase (GLUL) was higher with increasing MASLD severity. These enzymes catalyze the conversion of glutamine to glutamate and glutamate to glutamine, respectively. We did not observe any significant gene expression difference between participants with grade 1 steatosis without MASH (1w/oM) and those with MASH (1wM).

Circulating glucagon was positively associated with the expression of arginosuccinate synthase 1 (ASS1, rho = 0.27, *p* = 0.0148) and negatively associated with the expression of ornithine transcarbamylase (OTC, rho = −0.24, *p* = 0.0287), glutamine synthetase (GLUL, rho = −0.27, *p* = 0.0153), and glutamate dehydrogenase (GLUD1, rho = −0.24, *p* = 0.0336).

## 4 DISCUSSION

In this study, we aimed to investigate the association between circulating glutamate and MASLD. We studied a cohort of 150 patients, all living with obesity and free of T2D, separated into 5 groups according to MASLD severity. We observed that circulating glutamate was positively associated with visceral adipocyte hypertrophy, a marker of adipose tissue dysfunction, as well as the severity of MASLD. We also showed that hepatic expression of genes coding for enzymes implicated in glutamate metabolism are associated with MASLD severity.

Our study allowed us to confirm the previously reported association between MASLD severity and circulating glutamate, serine, tyrosine, and alanine (Tricò et al., 2021). We demonstrated for the first time that these associations persist when patients with and without MASLD are matched for BMI and T2D status. Interestingly, we did not find the circulating BCAAs to be different across study groups. This discrepancy with the literature could be because all our patients were living with obesity and were free of T2D, whereas most studies available did not control for these conditions (Tricò et al., 2021). Among patients with mild steatosis (grade 1), we did not observe a significant difference in amino acid levels between individuals with and without MASH (1w/oM vs. 1wM), indicating that the amino acid alterations observed in MASLD relate more closely to steatosis than to inflammation processes.

We did not find circulating glucagon to be different between our study groups. Higher glucagon levels were previously reported when comparing MASLD patients to controls in some (Wewer Albrechtsen et al., 2018), but not all studies (Eriksen et al., 2019; Pedersen et al., 2020). For example, Pedersen et al. reported no significant difference in glucagon levels between participants living with obesity with or without MASLD. In contrast, glucagon was significantly higher in both groups when compared to lean controls (Pedersen et al., 2020). This suggests that body weight, rather than MASLD, may be associated with elevated circulating glucagon levels. Additional studies carefully considering obesity and T2D are required to investigate the determinants of circulating glucagon levels. The association between circulating glucagon and hepatic mRNA levels not being significantly different between study groups (ASS1, OTC, and GLUD1) is intriguing. It indirectly suggests that glucagon and MASLD might both affect hepatic amino acid metabolism, but through different mechanisms. Additional studies would be required to investigate causality.

GLS1 and GLS2 are isoforms coding for the enzyme glutaminase. GLS1 is primarily expressed in the kidney, whereas GLS2 is mainly expressed in the liver (Matés et al., 2013). We did not observe differences in the hepatic expression level of GLS2 across groups, whereas we observed that GLS1 hepatic expression was higher with increasing MASLD severity. Interestingly, recent studies have reported hepatic overexpression of GLS1, but not GLS2, in the context of MASH (Simon et al., 2020) and hepatocarcinoma (Yu et al., 2015). This association between GLS1 and MASLD as well as  the functional implications of this phenomenon warrant investigations.

**FIGURE 7:**
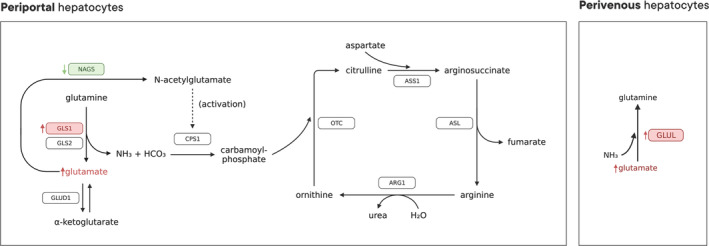
Schematic summary of the circulating amino acid and the hepatic gene expression results. Genes and amino acids in red were positively associated with MAFLD severity (higher in patients with more deteriorated livers). In contrast, genes and amino acids in green were inversely associated with MAFLD severity (lower in patients with more deteriorated livers). ARG1, arginase 1; ASL, arginosuccinate lyase; ASS1, arginosuccinate synthase 1; CPS1, carbomoyl‐phosphate synthetase 1; GLS1: glutaminase 1; GLS2, glutaminase 2; GLUD1, glutamate dehydrogenase 1; GLUL, glutamine synthetase; NAGS, N‐acetyl glutamate synthase; OTC, ornithine transcarbamylase.

The liver is highly compartmentalized in its functions; hepatocytes near the portal vein (periportal) express enzymes responsible for the liberation of ammonia from glutamine as well as the urea cycle enzymes (Hou et al., 2020). Hepatocytes near the central vein (perivenous) express an enzyme responsible for the addition of an amino group to glutamate to form glutamine. The function of this pattern is thought to be the recapture of ammonia not processed by the urea cycle to avoid increasing circulating ammonia levels (Eriksen et al., 2019). The results from our hepatic gene expression analysis indirectly suggest that MASLD severity may be associated with a greater liberation of ammonia from glutamine (higher GLS1 expression), reduced activation of the urea cycle (lower NAGS expression) as well as a recapture of ammonia (higher GLUL expression) (Figure 7). However, caution must always be used in interpreting mRNA results as they do not necessarily translate to changes in enzymatic activity. Although intriguing, the results presented here need to be validated with protein abundance and activity assays.

The strengths of this study are the detailed characterization of the participants, including liver and adipocyte histology, as well as the measurement of the hepatic expression of target genes. The limitations include the absence of a lean control group and the fact that we did not measure visceral adipose tissue area or volume with a gold‐standard method such as computed tomography. However, we did measure the size of visceral adipocytes, which has been reported to be closely correlated with visceral adipose tissue accumulation (Côté et al., 2016). Finally, the results of this study rely on correlations and cannot be used to assess the functional impact or the causality of the associations reported. Overall, we demonstrated that in individuals living with obesity without T2D, the severity of MASLD, mostly through steatosis and not steatohepatitis, is associated with higher circulating glutamate levels and differences in hepatic gene expression of enzymes involved in glutamate metabolism. This supports the hypothesis that changes in hepatic glutamate metabolism in the context of MASLD could contribute to the previously reported association between circulating glutamate and visceral obesity (Kimberly et al., 2017; Maltais‐Payette et al., 2018, 2019, 2022; Yamakado et al., 2012). However, the present study does not allow us to determine the causality of these associations which warrants further investigation.

## AUTHOR CONTRIBUTIONS

Conceptualization: IMP and AT. Data curation: IMP, JB, and FB. Formal analysis: IMP. Funding acquisition: AT and BJA. Investigation: LB, SM, FJ, and CC. Methodology: JB, PLM, CC, MFG, FB, and JC. Supervision: JC, BJA, and AT. Validation: AT and JB. Visualization: IMP. Writing—original draft: IMP and AT. Writing—review and editing: JB, MFG, LB, SM, FJ, PLM, CC, FB, JC, and BJA.

## FUNDING INFORMATION

This study was funded by research grants from the Canadian Institutes of Health Research and the Foundation of Quebec Heart and Lung Institute, respectively, to BJA and AT. IMP is the recipient of a scholarship from the Canadian Institutes of Health Research. JB is the recipient of a scholarship from the Fonds de recherche du Québec – Santé. LB and AT are co‐directors of the Research Chair in Bariatric and Metabolic Surgery at Laval University. JC acknowledges the support of the Canada Research Chair in Medical Genomics.

## CONFLICT OF INTEREST STATEMENT

AT and LB receive research funding from Johnson & Johnson, Medtronic, and GI Windows for studies on bariatric surgery. AT and LB acted as consultants for Bausch Health and Novo Nordisk. AT and LB are co‐directors of the Canada Research Chain in Bariatric and Metabolic Surgery at Laval University. AT is consultant for and received research funding from Biotwin. BJA is a consultant for Novartis, Editas Medicine, Eli Lilly, and Silence Therapeutics and has received research contracts from Pfizer, Eli Lilly, and Silence Therapeutics. JC is a general partner at Linearis.com.

## ETHICS STATEMENT

Tissue specimens and clinical data were obtained from the IUCPQ Biobank according to institutionally approved management modalities. This study was conducted in accordance with the declarations of Helsinki and Istanbul. Ethics approval was received on August 24th, 2020 (approval number 2021–3398, 21,925). All participants provided written informed consent.

## Data Availability

Anonymized data may be shared upon reasonable request to the corresponding author.
